# Supplementary material for: Content-rich biological network constructed by mining PubMed abstracts
Source: BMC Bioinformatics. 2004 Oct 8;5:147. doi: 10.1186/1471-2105-5-147 (PMC528731; doi:10.1186/1471-2105-5-147)
Supplement: Additional File 2 — The original results of the above study (non-essential files are deleted to keep the file size under the limit set by BMC bioinformatics). [file 1471-2105-5-147-S2.bz2 › chilibotAdditionalFile2/dip40/726ID9822594E18075/html/HLYD_HLYA.html]

 


 **HLYD** and **HLYA** 
  
Found 65 abstracts in PubMed, retrieved 40.  
 

 What does Google say? 
 PDF only 
| .edu only 

---

**Interactive relationship** (e.g. stimulation, inhibition, etc)

**Neutral relationship**- **HlyA**  remained in the periplasm unless the  **hlyD**  and tolC gene products were present in addition to the hybrid.  Ref: 1400227 J Bacteriol, 1992
- Our data suggest that TnhlyAs generated hemolysin  [ **HLYA** ]  fusion proteins are secreted efficiently by the HlyB  **HlyD**  TolC hemolysin  [ **HLYA** ]  secretion machinery.  Ref: 9711856 FEMS Microbiol Lett, 1998
- Our mutational analyses of the  **HlyA**  targetting signal and definition for the first time of stages and intermediates in the HlyB  **HlyD**  dependent translocation allow a discussion of the hemolysin  [ **HLYA** ]  export process in the wider context of protein translocation.  Ref: 1419114 FEMS Microbiol Immunol, 1992
- The secretion of the 107 kDa hemolysin  [ **HLYA** ]  A  **HlyA**  from Escherichia coli is mediated by membrane proteins hemolysin  [ **HLYA** ]  B HlyB and hemolysin  [ **HLYA** ]  D  **HlyD** .  Ref: 7703231 Biochemistry, 1995
- Selection for transport competence of C terminal polypeptides derived from Escherichia coli hemolysin  [ **HLYA** ]  the shortest peptide capable of autonomous HlyB  **HlyD**  dependent secretion comprises the C terminal 62 amino acids of  **HlyA** .  Ref: 7531275 Mol Gen Genet, 1994
- This sequence of  **HlyD**  also seems to interact with a component of the hemolysin  [ **HLYA** ]  secretion machinery since a hybrid  **HlyD**  protein carrying the corresponding TolC sequence, although inactive in the transport of  **HlyA** , is able to displace wild type  **HlyD**  from the secretion apparatus.  Ref: 7816028 Mol Gen Genet, 1994
- The system which consists of the two specific membrane proteins HlyB and  **HlyD**  and the outer membrane protein TolC, recognizes on  **HlyA**  a C terminally located signal sequence of about 60 amino acids.  Ref: 9382730 Behring Inst Mitt, 1997
- The last amino acid of  **HlyD** , Arg478, is possibly involved in the release of the  **HlyA**  protein, since cells bearing a  **hlyD**  gene mutant at this position produce similar amounts of  **HlyA**  to the wild type strain, but most of the protein remains cell associated.  Ref: 7816028 Mol Gen Genet, 1994
- Our results are compatible with the idea that a TolC, together with the TolC homologous part of  **HlyD** , forms a pore in the outer membrane through which hemolysin  [ **HLYA** ]  is released and vancomycin taken up.  Ref: 9393456 Mol Gen Genet, 1997
- Secretion of haemolysin  **HlyA**  is secA independent, but depends upon two accessory membrane proteins, HlyB and  **HlyD** , encoded by the hly determinant.  Ref: 1791766 Mol Microbiol, 1991
- The genetic determinants for a fusion of its B subunit StxB, which mediates toxin binding to target cells, with the COOH terminus of Escherichia coli hemolysin  [ **HLYA** ]  A Stx  **HlyA**  has been combined with the determinants of the accessory translocator proteins HlyB and  **HlyD**  in a Notl cassette.  Ref: 8905616 Microb Pathog, 1996
- The export of Escherichia coli hemolysin  [ **HLYA** ]  across the cytoplasmic and the outer membranes requires the COOH terminal signal sequence of  **HlyA** , the two specific translocator proteins HlyB and  **HlyD** , and the outer membrane protein TolC.  Ref: 9630987 Nat Biotechnol, 1996
- Secretion of Escherichia coli hemolysin  [ **HLYA** ]  is mediated by a sec independent pathway which requires the products of at least three genes, hlyB,  **hlyD**  and tolC.  Ref: 7816028 Mol Gen Genet, 1994
- A C terminal fragment 23 kDa of  **HlyA** , when fused at the C terminus, efficiently promoted the secretion of the eukaryotic protein prochymosin PCM to the medium via HlyB and  **HlyD** .  Ref: 1791766 Mol Microbiol, 1991
- We engineered plasmid pETR14, containing the hlyB and  **hlyD**  genes of the Escherichia coli hemolysin  [ **HLYA** ]  operon, to express a fusion protein containing 720 amino acid residues from the nontoxic, receptor binding, carboxy terminus of C. difficile toxin A and the secretion signal of E. coli hemolysin  [ **HLYA** ]  A.  Ref: 9199470 Infect Immun, 1997

**Non-interactive relationship** (e.g. studied together, co-existance, homology, etc.)

- This suggests that the capacity of the HlyB  **HlyD**  translocation system is high and not normally saturated by its natural  **HlyA**  substrate.  Ref: 2277639 Mol Gen Genet, 1990
- This PagC HlyAs fusion protein was expressed and efficiently secreted into the culture supernatant by E. coli upon complementation with the hemolysin  [ **HLYA** ]  secretion proteins HlyB and  **HlyD** .  Ref: 8766698 Appl Microbiol Biotechnol, 1996
- An Escherichia coli strain with wild type hemolysin  [ **HLYA** ]  secretion genes hlyB and  **hlyD**  and containing a cloned frpA gene secreted FrpA.  Ref: 8514394 Infect Immun, 1993
- Our data suggest that all beta Gal HlyAs fusion proteins generated are secreted, albeit with varying efficiencies, by the HlyB  **HlyD**  TolC hemolysin  [ **HLYA** ]  secretion machinery under Sec proficient conditions.  Ref: 8842146 Mol Gen Genet, 1996
- Transport of the  **HlyA**  derivatives by the HlyB  **HlyD**  system was compared with the wild type level and the data indicate that the  **HlyA**  C terminal export signal lies within the last 48 amino acids and comprises three functional domains an amphipathic, charged helix between residues 77 and R, 996.  Ref: 1791754 Mol Microbiol, 1991
- Secretion of  **HlyA**  occurs without a periplasmic intermediate and is directed by an uncleaved C terminal targetting signal and the HlyB and  **HlyD**  translocator proteins, the former being a member of a transporter superfamily central to import and export of a wide range of substrates by prokaryotic and eukaryotic cells.  Ref: 1365905 Bioessays, 1992
- Identification and characterization of two functional domains of the hemolysin  [ **HLYA** ]  translocator protein  **HlyD** .  Ref: 7816028 Mol Gen Genet, 1994
- However, hemolytic activity was found in the broth culture supernatant after transforming EDL 933 with the recombinant plasmid pRSC6 carrying the hlyB and  **hlyD**  genes from the E. coli alpha hemolysin  [ **HLYA** ]  operon.  Ref: 7868227 Infect Immun, 1995
- In order to identify stages and intermediates in this unconventional secretion process we have examined the effect of inhibition of the total proton motive force delta P and its components during the in vivo HlyB  **HlyD**  dependent export of a 22.4 kDa secretion competent  **HlyA**  C terminal peptide Actp.  Ref: 1915293 EMBO J, 1991
- Together with the inner membrane protein  **HlyD**  and the outer membrane protein TolC, HlyB is responsible for transport of the 107 kDa toxin  **HlyA**  from the cytoplasm, across both membranes of the cell envelope of Escherichia coli, directly to the medium.  Ref: 8366127 J Biol Chem, 1993
- This recombinant plasmid expressed a fusion protein Gcp F which was secreted into the culture supernatant by E. coli cells when the alpha hemolysin  [ **HLYA** ]  secretion functions HlyB and  **HlyD**  are supplied in trans.  Ref: 8150268 FEMS Microbiol Lett, 1994
- We show that an scFv  **HlyA**  hybrid secreted by Hly type I transporter TolC, HlyB,  **HlyD**  is accumulated in the extracellular medium with the disulphide bonds correctly formed.  Ref: 11309117 Mol Microbiol, 2001
- The alternative secretion pathway which exports hemolysin  [ **HLYA** ]  across both Escherichia coli membranes into the surrounding medium is directed by an uncleaved C terminal targeting signal and the membrane translocator proteins  **HlyD**  and HlyB.  Ref: 1915293 EMBO J, 1991
- Using a dual plasmid complementation system, we show that the alpha hemolysin  [ **HLYA** ]  hlyB and  **hlyD**  transport determinants are sufficient to allow secretion and activation of a functional metalloprotease species from E. coli, as are the comparable protease secretion functions of Erwinia chrysanthemi.  Ref: 1551853 J Bacteriol, 1992
- We studied the efficiency of the pHly152 derived haemolysin transport system using PhoA  **HlyA**  fusion proteins and different constructs which provide HlyB  **HlyD**  in trans.  Ref: 2277639 Mol Gen Genet, 1990
- Extracellular secretion of  **HlyA**  by the TetC HlyB hybrid required  **HlyD**  and TolC.  Ref: 1400227 J Bacteriol, 1992
- The structure and function of the components of the  **HlyA**  secretion apparatus, HlyB,  **HlyD**  and TolC, have been studied in great detail.  Ref: 11755084 Trends Microbiol, 2002
- A 10.2 kb fragment that encoded hemolytic activity in the phage lysate was aligned by Southern blot hybridization to genes hlyC,  **hlyA** , hlyB, and  **hlyD**  of the Escherichia coli hemolysin  [ **HLYA** ]  operon, and expression of the A. pleuropneumoniae genes in E. coli revealed that they have the same functions as their E. coli analogs hlyIC encodes a protein that activates inactive 105 kDa prohemolysin I encoded by hlyIA to active hemolysin  [ **HLYA** ]  I, while hlyIB and hlyID are necessary for HlyIA secretion.  Ref: 1639473 Infect Immun, 1992
- A LacZ HlyAs, fusion protein carrying, at the C terminus of LacZ, the 60 amino acid signal sequence of  **HlyA**  was not secreted in the presence of HlyB  **HlyD** .  Ref: 1552901 Mol Gen Genet, 1992
- To this end, Salmonella typhimurium SL7207 was used as carrier for secreted p60  **HlyA**  fusion protein by Escherichia coli HlyB and  **HlyD**  transport proteins.  Ref: 7729919 Infect Immun, 1995
- We have developed an export cloning system that is composed of two vectors one in which the fusion of the desired gene with the 3 end of  **hlyA**  is generated, and a second in which the sequences containing the fusion are combined with the accessory genes hlyB and  **hlyD** , thereby reconstructing the natural organization of the hly locus.  Ref: 9630987 Nat Biotechnol, 1996
- It was also found that the CvaA component of the colicin V export system shows amino acid sequence similarities with another component involved in hemolysin  [ **HLYA** ]  export,  **HlyD** .  Ref: 2249654 EMBO J, 1990
- The toxin  **HlyA**  is exported from Escherichia coli, without a periplasmic intermediate, by a type I system comprising an energized inner membrane IM translocase of two proteins,  **HlyD**  and the traffic ATPase HlyB, and the outer membrane OM porin like TolC.  Ref: 9822594 EMBO J, 1998
- Production of active Serratia marcescens metalloprotease from Escherichia coli by alpha hemolysin  [ **HLYA** ]  HlyB and  **HlyD** .  Ref: 1551853 J Bacteriol, 1992
